# Does perioperative allogeneic blood transfusion worsen the prognosis of patients with hepatocellular carcinoma? A meta-analysis of propensity score-matched studies

**DOI:** 10.3389/fonc.2023.1230882

**Published:** 2023-10-02

**Authors:** Lingbo Hu, Zhenyu Li, Yingli Qiao, Aidong Wang

**Affiliations:** ^1^ Department of Hepatopancreatobiliary Surgery, Taizhou Hospital of Zhejiang Province Affiliated to Wenzhou Medical University, Taizhou, Zhejiang, China; ^2^ Department of Hepatopancreatobiliary Surgery, Enze Hospital, Taizhou Enze Medical Center (Group), Taizhou, Zhejiang, China

**Keywords:** liver resection, blood transfusion, allogeneic, hepatocellular carcinoma, prognosis

## Abstract

**Background:**

Allogeneic blood transfusion is required in a part of liver resection. The effect of allogeneic blood transfusion on the prognosis of patients with hepatocellular carcinoma (HCC) remains controversial. To investigate whether perioperative allogeneic blood transfusion (PBT) affects the long-term prognosis of patients with HCC, we conducted a meta-analysis that included only propensity score-matched (PSM) studies.

**Methods:**

The Cochrane Library, Embase, PubMed, and Web of Science databases were systematically searched to identify PSM studies that compared the long-term outcomes of allogeneic blood transfusion in resected HCC patients. Overall survival (OS) and recurrence-free survival (RFS) rates were calculated.

**Results:**

This meta-analysis included 9 PSM studies with 12 datasets involving 2476 patients. Lower OS and RFS in HCC patients receiving allogeneic blood transfusion were observed than those in patients not receiving blood transfusion (OS: hazard ratio [HR], 1.34; 95% confidence interval [CI], 1.10–1.64; p < 0.01; RFS: HR, 1.29; 95% CI, 1.07–1.56; p < 0.01). Subgroup analysis revealed that among patients with BCLC A HCC, those receiving allogeneic blood transfusion had lower OS and RFS (OS: HR, 2.27; 95% CI, 1.61–3.21; RFS: HR, 2.11; 95% CI, 1.30–3.41). OS and RFS were similar in both groups of patients with BCLC B and C HCC.

**Conclusion:**

The receipt of perioperative allogeneic blood transfusion is associated with a decrease in OS and RFS. These results seem to be reliable for patients in BCLC stage A. But more high-quality research is needed to confirm this conclusion.

## Introduction

Liver cancer is the third most common cause of cancer-related death worldwide, and hepatocellular cancer (HCC) accounts for most primary liver cancer cases (1). Liver resection (LR) is a common treatment approach for HCC (2), and it is associated with greater intraoperative blood loss that requires blood transfusion (3). The early adverse events of blood transfusion included transfusion reactions, transfusion‐related acute lung injury, transfusion‐related circulatory overload, anaphylaxis, and transmission of blood‐borne pathogens. As for the effect on long-term survival, one reason is the impact of blood transfusions on the immune system. Blood transfusion is considered to induce immunosuppression by reducing natural killer (NK) cell activity and increasing suppressor T lymphocyte activity (4). In a previous study, the decreased levels of T lymphocytes, NK cells, and immunoglobulins after allogeneic blood transfusion were related to a higher tumor recurrence rate in HCC patients (5).

Several early meta-analyses have revealed the negative effect of allogeneic blood transfusion on the long-term survival of patients with HCC (6, 7). However, this conclusion remains controversial because these meta-analyses were based on nonrandomized studies with significant confounding or selection bias. Recently, several propensity score-matched (PSM) studies conducted on this topic have reported contradictory conclusions (8–16). Therefore, to clarify the effect of perioperative allogeneic blood transfusion on the long-term prognosis of patients with HCC, we conducted the present meta-analysis that included only PSM studies.

## Methods

This systematic review is registered in PROSPERO (registration no. CRD42023426321).

### Search strategy

Four databases, namely PubMed, Embase, Web of Science, and Cochrane Library, were searched for English articles related to the studied topic from conception to April 16, 2023. The following keywords and MeSH terms were used in PubMed search: hepatocellular carcinoma, hepatectomy, hepatic resection, liver resection, and blood transfusion. The details of the search strategy for all databases are shown in **Supplementary Material S1**. References in the identified studies were further searched manually for additional relevant studies.

### Inclusion criteria

The inclusion criteria were as follows: (1) articles that reported randomized controlled trials (RCTs) and PSM studies and were published in English; (2) studies that used LR with or without allogeneic blood transfusion for treating HCC patients; and (3) studies that reported overall survival (OS) and/or recurrence-free survival (RFS).

### Exclusion criteria

The following types of studies were excluded: (1) studies that focused on autologous transfusion and (2) noncomparative and non-PSM studies, abstracts, case reports, and reviews. Moreover, for studies with overlapped patient cohorts, only the top study (highest quality, largest sample size, or the most recent one) was included, and the remaining studies were excluded.

### Definition

OS was defined as the time between surgery and death and RFS was defined as the time from surgery to tumor recurrence. Herein, OS and RFS were considered as primary time-to-event outcomes. 1-, 3-, and 5-years overall survival rates and recurrence-free survival rates were defined as the percent of patients survival or without tumor recurrence at 1, 3, and 5 years after liver resection.

### Quality assessment and data extraction

The initial quality assessment of each study and the subsequent data extraction were conducted independently by two researchers (LB Hu and XP Shi). For nonrandomized comparative trials, the Newcastle-Ottawa scale (NOS) with a score of up to 9 points (5 or less for low quality; 6–7 for medium quality; and 8 or more for high quality) was used for quality assessment (17).

Predesigned and standardized forms were used to extract the study details from the included studies (e.g., first author, year of publication, patient information, and tumor characteristics). The primary outcomes, including OS, RFS, survival rates (1-, 3-, and 5-year), and RFS rates, were extracted either directly from the original reports or indirectly by estimation with the Kaplan-Meier curve using the Engauge Digitizer software (version 4.1) based on the approach introduced by Tierney et al. (18, 19). Any disagreements between the two independent researchers were resolved by a third researcher (AD Wang).

### Statistical analysis

The inverse variance method was used to determine the hazard ratio (HR) and 95% confidence interval (CI) values. The Mantel-Haenszel method was used to estimate the risk ratio (RR) and 95% CI values. Heterogeneity was assessed using the χ^2^ method (I^2^ values of 25% and 50% indicated low heterogeneity and moderate heterogeneity, respectively). The test model was selected based on the heterogeneity level, and the random-effects model was used for studies with I^2^ > 50% (20). The robustness of the conclusion was determined by sensitivity analysis. Subgroup analysis was based on the Barcelona Clinic Liver Cancer (BCLC) staging system and the balance in the volume of blood loss between the two groups. The publication bias was assessed using funnel plots with Begg’s test and Egger’s test. A p-value of <0.05 was considered to indicate statistical significance. All statistical analyses were conducted in R program (version 4.2.3).

## Results

### Study search and selection details

**Figure 1:**
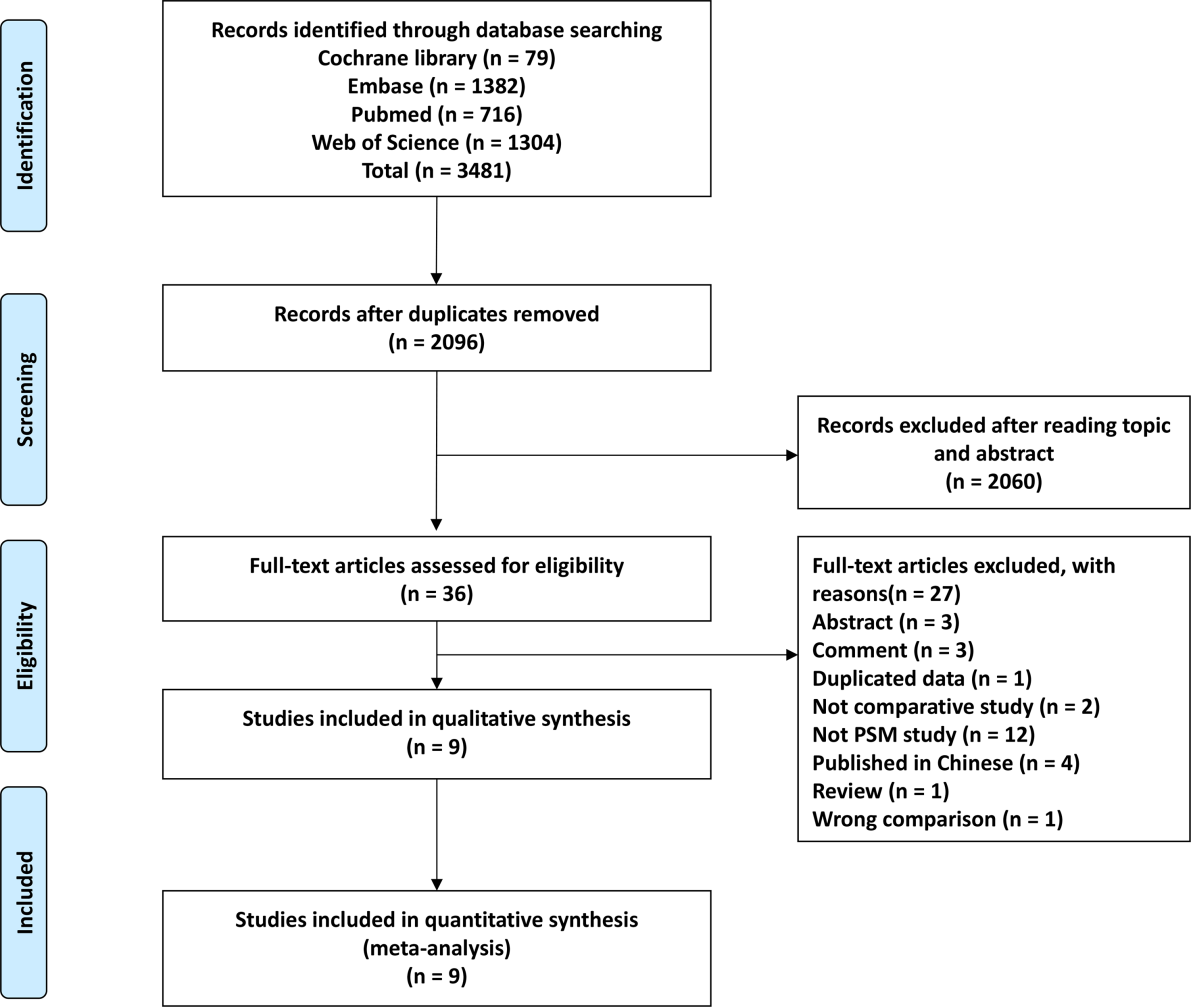
Flow chart of the study selection process.

The database search yielded 2,096 articles, of which 2,060 articles were excluded after reviewing the title and abstract (**Figure 1**). Among the remaining articles, 27 articles did not meet the inclusion criteria and were therefore excluded. Thus, 9 studies were selected for the meta-analysis (8–16).

### Study characteristics

All the 9 included studies with 12 datasets were PSM studies including 8028 patients, of which 21.50% of patients (1726) received perioperative allogeneic blood transfusion, and the remaining 78.50% of patients did not receive perioperative allogeneic blood transfusion. After the PSM approach with 1:1 match, the includded studies involved 2476 patients, of which 50% patients (1238) received perioperative allogeneic blood transfusion (the PBT group), and the remaining 50% patients did not receive perioperative allogeneic blood transfusion (the NPBT group). Patient characteristics are shown in **Table 1**. The median volume of blood loss ranged from 400 to 1870 mL in the PBT group and from 200 to 1200 mL in the NPBT group. The volume of blood loss between the PBT and NPBT groups was balanced in 6 studies (8, 10–12, 15, 16) and not balanced in 3 studies (9, 13, 14). Three studies assessed the effect of blood transfusion on long-term prognosis by stratifying the patients according to the tumor stage (10–12). Four studies excluded patients died in early period post operation (30-day, 90-day, or in-hospital) (9, 11, 12, 15), while other studies included these patients.

**Table 1:** Characteristics of the included studies.

Study	Group	Sample size	Age (year)	Gender M/F	HBV/HCV	Liver cirrhosis	AFP ng/mL	Child-Pugh A/B	Tumor size cm	Tumor number S/MU	Histological grading 1 + 2/3 + 4	MVI +	Blood loss mL	Blood transfusion mL	Hepatectomy major/minor	Resection margin
Tan 2023 (8)	PBT	163	65 (58–73)^ζ^	130/33	61/89	78	60 (>200) 103 (<200)	148/15	5.0 (3.0–9.0)^ζ^	35/127	90/67	46	800 (500–1000)^ζ^	500 (350‐600)^ζ^	69/94	12 (positive)
	NPBT	163	66 (58–72)^ζ^	133/30	70/81	79	67 (>200) 196 (<200)	149/14	5.0 (3.2–9.0)^ζ^	40/122	101/56	51	600 (500–1000)^ζ^		70/93	8 (positive)
Nakayama 2023 (9)	PBT	133	69 (40–85)^η^	98/35	15/81	59	35.3 (0.8–117,800)^η^	127/6	3.8 (1.0–18.0)^η^	89/44	NA	37	660 (10–2,065)^η^	NA	15/118	2.0 cm (0–29)^η^
	NPBT	133	69 (32–86)^η^	99/34	12/87	60	15.8 (1.3–93,075)^η^	126/7	3.5 (0.2–18.0)^η^	90/43	NA	27	616 (15–2,231)^η^		14/119	1.0 cm (0–40)^η^
Xia 2022 (10)	PBT	82	46.3 ± 10.7	74/8	67/3	NA	37 (≤400) 45 (>400)	59/23	6.2 (4.9–7.3)^η^	79/3	31/51	3	700 (250,1600)^η^	NA	26/56	NA
Stage A	NPBT	82	49.6 ± 11.6	70/12	64/3	NA	45 (≤400) 37 (>400)	71/11	6.1 (4.7–9.4)^η^	79/3	39/43	0	400 (200,1250)^η^		29/53	NA
Xia 2022 (10)	PBT	57	45.4 ± 9.8	51/6	51/3	NA	12 (≤400) 45 (>400)	51/6	10.5 (7.2–14.9)^η^	36/21	30/27	18	750 (250,2000)^η^	NA	25/32	NA
Stage B + C	NPBT	57	44.6 ± 10.3	57/0	51/3	NA	12 (≤400) 45 (>400)	45/12	10.1 (7.0–12.1)^η^	36/21	27/30	21	400 (200,1,700)^η^		22/35	NA
Peng 2021 (11)	PBT	53	54.9 ± 10.9	45/8	47 (HBV+HCV)	44	25.0 (6.0–377.0)^ζ^	51/2	4.0 (3.0–6.0)^ζ^	53/0	39/14	1	0.8 L (0.5 - 1.2)^ζ^	NA	12/41	14 (<1 cm)
Stage A	NPBT	53	53.0 ± 10.8	48/5	51 (HBV+HCV)	42	16.0 (4.6–203.5)^ζ^	52/1	4.0 (3.0–6.0)^ζ^	53/0	42/11	2	0.2 L (0.1 - 0.4)^ζ^		11/42	20 (<1 cm)
Peng 2021 (11)	PBT	51	54.9 ± 10.1	41/10	41 (HBV+HCV)	41	98.0 (6.0–1784.0)^ζ^	51/0	5.0 (4.0–7.0)^ζ^	35/16	32/19	25	1.0 L (0.6 - 1.3)^ζ^	NA	23/28	20 (<1 cm)
Stage B + C	NPBT	51	53.6 ± 9.9	44/7	45 (HBV+HCV)	42	86 (6.0–837.0)^ζ^	51/0	5.0 (4.0–8.0)^ζ^	39/12	27/24	24	0.3 L (0.2 - 0.5)^ζ^		23/28	16 (<1 cm)
Chen 2020 (12)	PBT	156	52.7 ± 12.3	127/29	132/NA	144	64 (≥400) 92 (<400)	NA	8.4 ± 4.4	153/3	108/48	35	1000 (50, 12,000)^η^	NA	59/97	51 (≤1 cm)
Stage A	NPBT	161	53.1 ± 12.4	126/35	138/NA	121	60 (≥400) 101 (<400)	NA	7.69 ± 3.86	156/5	111/50	33	200 (50,3000)^η^		58/103	45 (≤1 cm)
Chen 2020 (12)	PBT	132	49.2± 12.6	118/14	112/NA	101	61(≥ 400) 71 (<400)	NA	9.8 ± 3.5	38/94	88/44	62	1000 (15,7000)^η^	NA	91/41	99 (≤1 cm)
Stage B + C	NPBT	127	51.4 ± 13.1	115/12	103/NA	93	70 (≥400) 57 (<400)	NA	10.1 ± 3.5	45/82	81/46	66	300 (50,2500)^η^		83/44	100 (≤1 cm)
Yamashita 2019 (13)	PBT	43	66 ± 10	38/5	16/19	NA	6368 ± 3517	43/0	4.4 ± 2.9	2 ± 2	NA	18	1006 ± 576	NA	12/31	NA
	NPBT	43	64 ± 10	40/3	16/18	NA	751 ± 3259	43/0	4.3 ± 2.7	2 ± 1	NA	14	1000 ± 514		15/28	NA
Wada 2018 (14)	PBT	74	53 (≥65) 21 (<65)	62/12	10/51	33	NA	68/6	4.3 ± 3.1	17/57	8/66	24	1223 ± 614	NA	NA	NA
	NPBT	74	45 (≥65) 29 (<65)	60/14	17/43	28	NA	67/7	4.3 ± 2.6	21/53	6/68	32	1200 ± 691		NA	NA
Yang 2016 (15)	PBT	234	49.7 ± 11.7	202/32	214/4	172	123 (≤400) 111 (>400)	200/34	66 (≤5) 168 (>5)	150/84	33/201	173	NA	NA	115/119	124 (≤1 cm)
	NPBT	234	50.1 ± 10.7	204/30	213/7	179	124 (≤400) 110 (>400)	200/34	67 (≤5) 167 (>5)	153/81	26/208	171	NA		110/124	114 (≤1 cm)
Kuroda 2012 (16)	PBT	60	59.4 (34–79)^η^	50/10	NA/38	30	138.3 (5–161774)^η^	46/14	61.6 mm (7–193)^η^	2.5 (1–10)^η^	1/59	35	1870 (30–7000)^η^	840 (280–6720)^η^	15/45	NA
	NPBT	60	60.7 (23–79)^η^	48/12	NA/44	35	70.1 (0.5–449860)^η^	43/17	60.6 mm (14–155)^η^	2.8 (1–10)^η^	3/57	31	459 (20–1500)^η^		12/48	NA

Stage A, BCLC stage A; Stage B and C, BCLC stage B and C; PBT, perioperative blood transfusion; NPBT, no perioperative blood transfusion; M, male; F, female; HBV, hepatitis B virus; HCV, hepatitis C virus; NA, not available; AFP, alfa fetoprotein; S, solitary; MU, multiple; MVI, microscopic vascular invasion.

### Quality assessment

The details of the quality assessment of the included studies are shown in **Supplementary Material S2**. Studies with a balance in the volume of blood loss between the PBT and NPBT groups scored 9 points, and those with no balance in the volume of blood loss between both groups scored 8 points. Thus, all studies were considered high-quality studies.

### Overall survival

**Figure 2:**
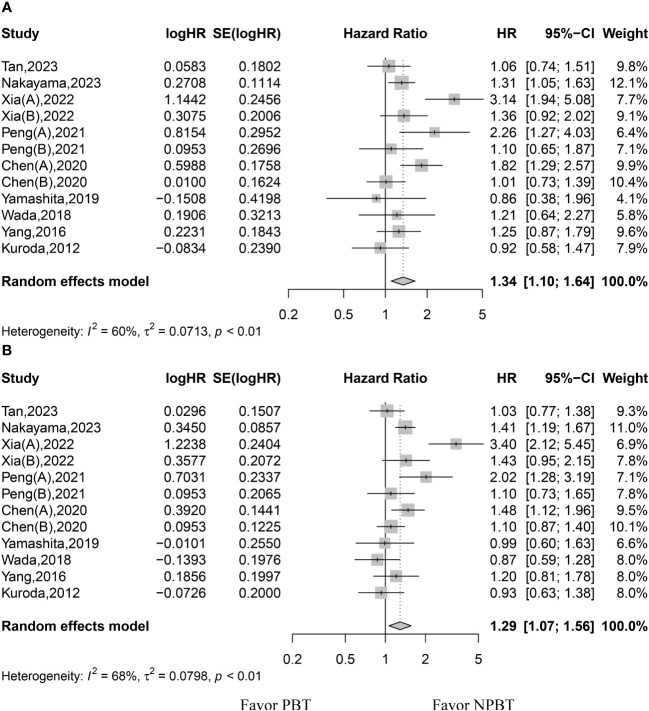
Forest plot for hazard ratios of overall survival (OS) and recurrence-free survival (RFS). **(A)** forest plot for OS. **(B)** forest plot for RFS. PBT, perioperative blood transfusion; NPBT, no perioperative blood transfusion.

The HR values of OS were available for all studies. The random-effects model was used because of significant heterogeneity among the studies. The pooled data showed that patients in the PBT group had lower OS (HR, 1.34; 95% CI, 1.10–1.64; p < 0.01) (**Figure 2**). The 1-, 3-, and 5-year survival rates were reported in 4, 5, and 9 studies, respectively. Because the studies showed significant heterogeneity in the 3- and 5-year survival rates, the random-effects model was used to pool the results from these studies. Patients in the PBT group had lower 1-, 3-, and 5-year survival rates (RR, 0.89; 95% CI, 0.84–0.96; p < 0.01; RR, 0.7839; 95% CI, 0.6150–0.9992; p = 0.0492; RR, 0.70; 95% CI, 0.51–0.96; p = 0.026, respectively) (**Table 2**).

**Table 2:** Details of overall survival and recurrence-free survival rates.

Outcome	No. of datasets	RR (95% CI)	p-value	I^2^	Model
1-year OS rate	4	0.89 (0.84–0.96)	0.0011	28.0%	Fixed
3-year OS rate	5	0.7839 (0.6150–0.9992)	0.0492	66.2%	Random
5-year OS rate	9	0.70 (0.51–0.96)	0.0260	72.9%	Random
1-year RFS rate	4	0.75 (0.51–1.11)	0.1475	83.8%	Random
3-year RFS rate	5	0.70 (0.41–1.20)	0.1927	81.2%	Random
5-year RFS rate	8	0.63 (0.39–1.02)	0.0583	68.9%	Random

OS, overall survival; RFS, recurrence-free survival; RR, risk ratio; CI, confidence interval.

### Recurrence-free survival

The HR values of RFS were available for all studies. The random-effects model was used because of significant heterogeneity among the studies. The pooled data showed that patients in the PBT group had lower RFS rates (HR, 1.29; 95% CI, 1.07–1.56; p < 0.01) (**Figure 2**). The 1-, 3-, and 5-year RFS rates were reported in 4, 5, and 8 studies, respectively. Because the studies showed significant heterogeneity in 1-, 3-, and 5-year RFS rates, the random-effects model was used to pool the results from these studies. Patients in both groups showed similar 1-, 3-, and 5-year RFS rates (RR, 0.75; 95% CI, 0.51–1.11; p = 0.1475; RR, 0.70; 95% CI, 0.41–1.20; p = 0.1927; RR, 0.63; 95% CI, 0.39–1.02; p = 0.0583, respectively) (**Table 2**).

### Sensitivity analysis and publication bias

The sensitivity analysis revealed good robustness of OS and RFS results (**Supplementary Material S3**). Funnel plots of the OS and RFS data combined with Begg’s test and Egger’s test indicated no significant publication bias (**Supplementary Material S4**).

### Subgroup analysis

The details of the subgroup analysis are shown in **Supplementary Material S5-7**. For patients with BCLC A HCC, those in the PBT group had lower OS and RFS rates (HR, 2.27; 95% CI, 1.61–3.21; HR, 2.11; 95% CI, 1.30–3.41, respectively). The OS and RFS rates in both groups were similar for patients with BCLC B and C HCC (**Supplementary Material S5**). Regarding the balance in the volume of blood loss between the two groups in the included studies, subgroup analysis showed better OS and RFS rates for the NPBT group regardless of balance or no balance in the volume of blood loss between the two groups (**Supplementary Material S6**). Subgroup analysis of studies excluded the patients died in early period post operation showed that those patients in the PBT group had lower OS and RFS rates (HR, 1.35; 95% CI, 1.10–1.67; HR, 1.33; 95% CI, 1.16–1.53, respectively), however, subgroup analysis of studies included the patients died in early period post operative showed that patients in both groups had similar OS and RFS.

## Discussion

The present meta-analysis of only PSM studies indicated that patients with HCC receiving perioperative allogeneic blood transfusion had poorer OS and RFS rates than those not receiving blood transfusion. Subgroup analysis showed that this outcome was consistent in HCC patients with BCLC stage A, while it was inconsistent in patients with BCLC stage B and stage C HCC. Additionally, the 1-, 3-, and 5-year OS rates were better in patients without blood transfusion, while the 1-, 3-, and 5-year RFS rates were similar in both groups of patients.

Theoretically, allogeneic blood transfusion leads to immunosuppression (4), and HCC recurrence has been reported to occur more frequently in immunosuppressed patients (5). A previous meta-analysis published in 2013 that included 22 studies involving 5635 patients demonstrated that patients receiving allogeneic blood transfusion had lower survival rates but higher recurrence and complication rates (7). However, a limitation of this meta-analysis was that both comparative and noncomparative studies were included, resulting in a lower confidence of evidence. Xun et al. conducted another meta-analysis that included 29 cohort studies without any language limitation (6). The authors showed that patients in the PBT group had lower 1-, 3-, 5-, and 10-year OS and disease-free survival rates. However, the baseline characteristics of patient and tumor were not balanced in included studies (21–23). Moreover, time-to-event outcomes are most appropriately analyzed using HR (19), while odds ratio or RR was used in the two above-mentioned meta-analyses.

To the best of our knowledge, the present meta-analysis of only PSM studies is the first to assess the effect of perioperative allogeneic blood transfusion on the long-term prognosis of patients with HCC. Currently, there is a lack of RCTs focused on this topic; hence, a meta-analysis of only PSM studies can be considered the highest level of evidence. The present meta-analysis also has some limitations. First, because a PSM study is essentially a retrospective study, selection bias is inevitable. Second, even after propensity score matching, the baseline blood loss was not balanced in several studies. Hence, we conducted a subgroup analysis to determine the effect of the balance of the baseline blood loss on the results. The negative effect of perioperative allogeneic blood transfusion on HCC prognosis remained consistent regardless of whether the baseline blood loss was balanced or not balanced. Third, significant heterogeneity was observed among the studies. However, sensitivity analysis indicated that the results were robust with no publication bias. Subgroup analysis showed that HCC patients with different BCLC stages was one of the sources of heterogeneity. Whether patients with early post operative mortality were included or excluded was another source of heterogeneity.

## Conclusion

The receipt of perioperative allogeneic blood transfusion is associated with a decrease in OS and RFS. Although, the RR for OS and RFS is significant, the confidence interval is close to 1 for most observations. So we have to be careful in our conclusions that blood transfusion reduces OS and RFS. Additional well-designed RCTs based on tumor stages are required to clarify the effect of blood transfusion on the prognosis of HCC patients with different BCLC stages.

## Data availability statement

The raw data supporting the conclusions of this article will be made available by the authors, without undue reservation.

## Author contributions

AW designed this work. LH and ZL extracted, analyzed and interpreted the data. ZL and YQ wrote a part of the manuscript. LH was a major contributor in writing the manuscript. All authors contributed to the article and approved the submitted version.
