# Supplementary material for: Does perioperative allogeneic blood transfusion worsen the prognosis of patients with hepatocellular carcinoma? A meta-analysis of propensity score-matched studies
Source: Front Oncol. 2023 Oct 2;13:1230882. doi: 10.3389/fonc.2023.1230882 (PMC10581339; doi:10.3389/fonc.2023.1230882)

Supplementary materials S1 Search strategy

| Databases | Search strategy |
| --- | --- |
| Pubmed | #1 ((hepatocellular carcinoma[MeSH Terms]) OR (hepatocellular carcinoma)) OR (HCC)  #2 (blood transfusion[MeSH Terms]) OR (blood transfusion)  #3 ((((hepatectomy[MeSH Terms]) OR (liver resection)) OR (hepatic resection)) OR (hepatectomy)) OR (surgical resection)  #4 #1 AND #2 AND #3 |
| Embase | #1 'liver cell carcinoma'/exp OR 'hepatocellular carcinoma':ti,ab,kw OR hcc:ti,ab,kw  #2 'hepatectomy'/exp OR 'liver resection':ti,ab,kw OR 'hepatic resection':ti,ab,kw OR hepatectomy:ti,ab,kw OR 'surgical resection':ti,ab,kw  #3 'blood transfusion'/exp OR 'blood transfusion':ti,ab,kw  #4 #1 AND #2 AND #3 |
| Cochrane Library | #1 MeSH descriptor: [Carcinoma, Hepatocellular]  #2 (hepatocellular carcinoma):ti,ab,kw OR (HCC):ti,ab,kw  #3 #1 OR #2  **#4 MeSH descriptor:[Hepatectomy] explode all trees**  **#5 (liver resection) :ti,ab,kw OR (hepatic resection) :ti,ab,kw OR (hepatectomy) :ti,ab,kw OR (surgical resection) :ti,ab,kw**  #6 #4 OR #5  #7 **MeSH descriptor:[Blood Transfusion] explode all trees**  #8 **(blood transfusion) :ti,ab,kw**  **#9 #7 OR #8**  **#10 #3 AND #6 AND #9** |
| Web of Science | **#1 ALL=** (hepatocellular carcinoma) OR ALL=HCC  #2 ALL= (blood transfusion)  #3 ALL= (liver resection) OR ALL= (hepatic resection) OR ALL= (hepatectomy) OR ALL= (surgical resection)  #4 #1 AND #2 AND #3 |

Supplementary material S2 NOS score for PSM studies

| Study | Selection | | | | Comparability | Outcome | | | Total score |
| --- | --- | --- | --- | --- | --- | --- | --- | --- | --- |
|  | Representativeness  of the  exposed cohort | Selection of  the non- exposed cohort | Ascertainment  of exposure | Demonstration  of outcome |  | Assessment of outcome | Follow-up was long enough | Adequacy  of follow up |  |
| Tan | * | * | * | * | * | * | * | * | 8 |
| Nakayama | * | * | * | * | ** | * | * | * | 9 |
| Xia(A) | * | * | * | * | * | * | * | * | 8 |
| Xia(B) | * | * | * | * | * | * | * | * | 8 |
| Peng(A) | * | * | * | * | * | * | * | * | 8 |
| Peng(B) | * | * | * | * | * | * | * | * | 8 |
| Chen(A) | * | * | * | * | * | * | * | * | 8 |
| Chen(B) | * | * | * | * | * | * | * | * | 8 |
| Yamashita | * | * | * | * | ** | * | * | * | 9 |
| Wada | * | * | * | * | ** | * | * | * | 9 |
| Yang | * | * | * | * | * | * | * | * | 8 |
| Kuroda | * | * | * | * | * | * | * | * | 8 |
|  |  |  |  |  |  |  |  |  |  |

Supplementary material S3 Forest plot for sensitivity analysis of overall survival and recurrence-free survival.

A, forest plot for sensitivity analysis of overall survival. B, forest plot for sensitivity analysis of recurrence-free survival.


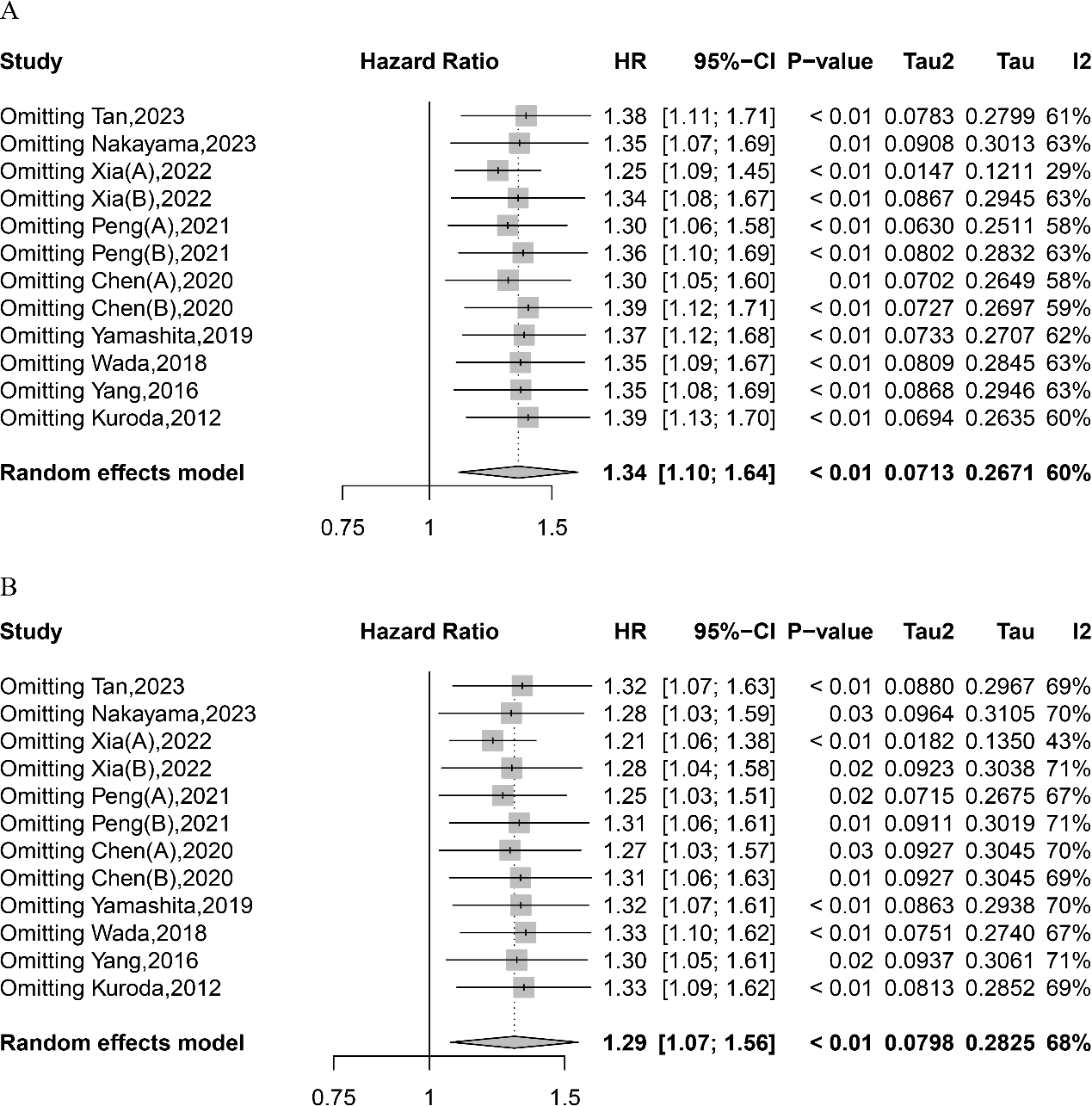


Supplementary material S4 Funnel plot for overall survival and recurrence-free survival.

A, funnel plot for overall survival. B, funnel plot for recurrence-free survival.


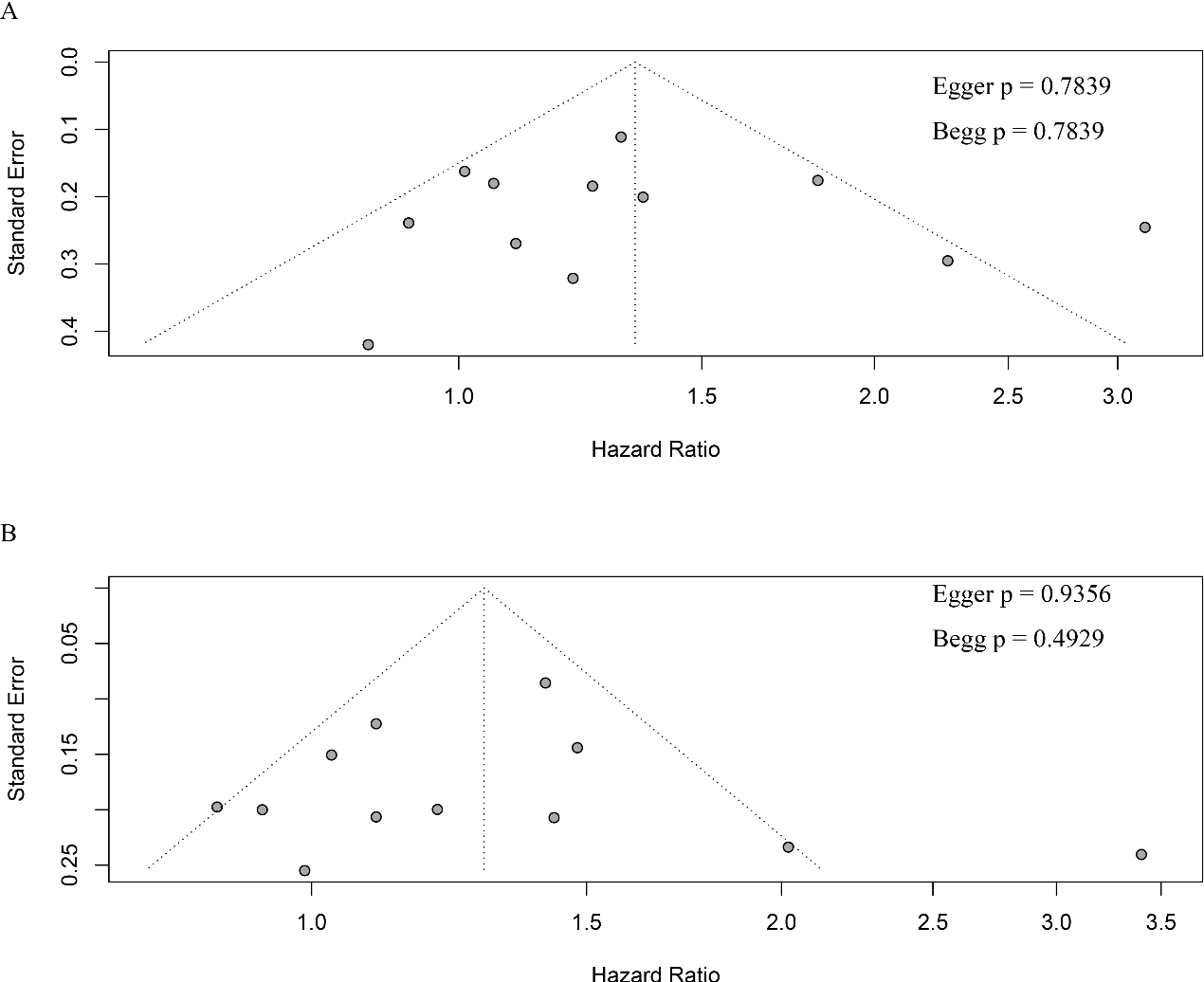


Supplementary material S5 Subgroup analysis based on BCLC stage for overall survival and recurrence-free survival.

A, subgroup analysis based on BCLC stage for overall survival; B, subgroup analysis based on BCLC stage for recurrence-free survival.
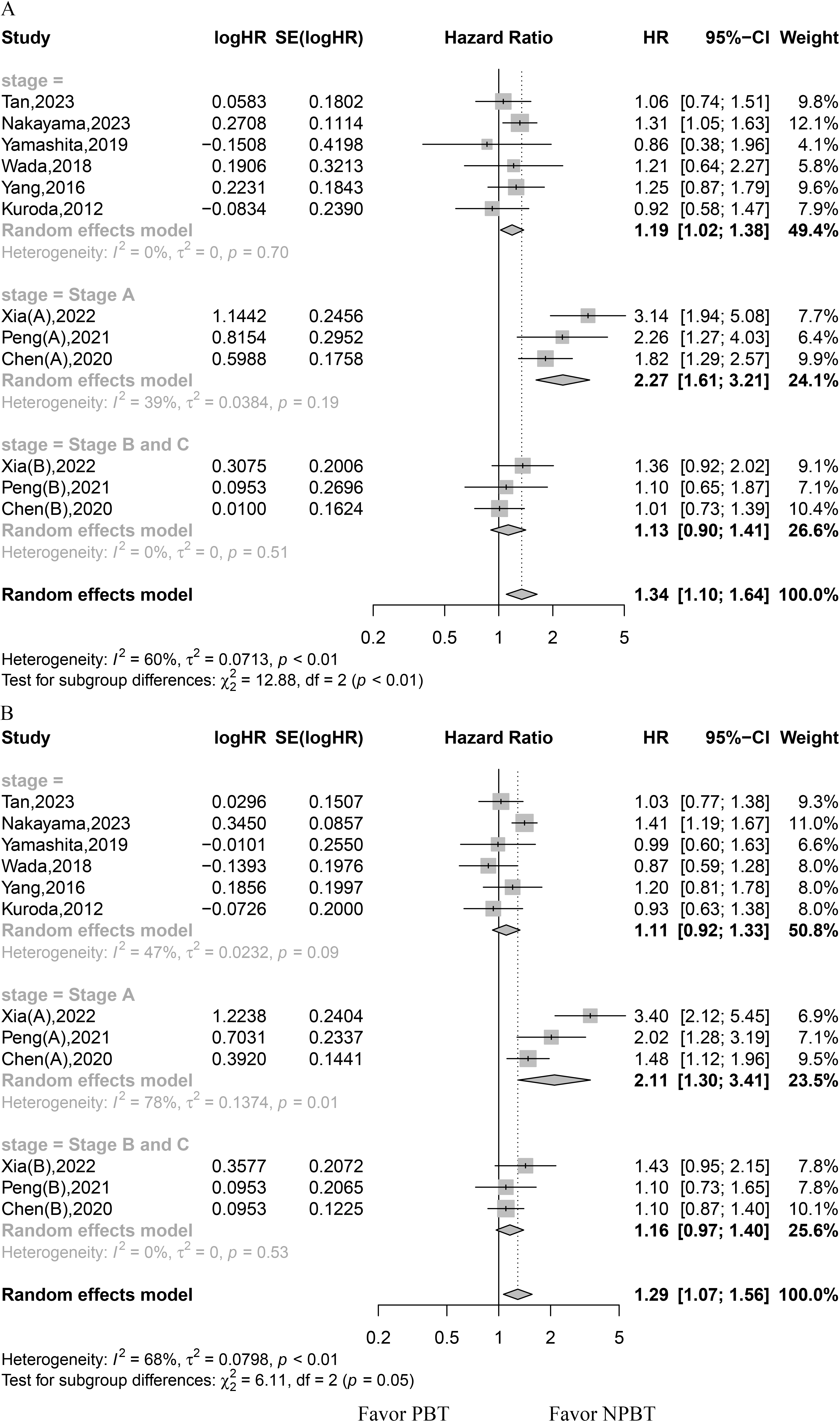


Supplementary material S6 Subgroup analysis based on the balance of blood loss between two groups in included studies for overall survival and recurrence-free survival.

A, subgroup analysis based on the balance of blood loss between two groups in included studies for overall survival; B, subgroup analysis based on the balance of blood loss between two groups in included studies for recurrence-free survival.


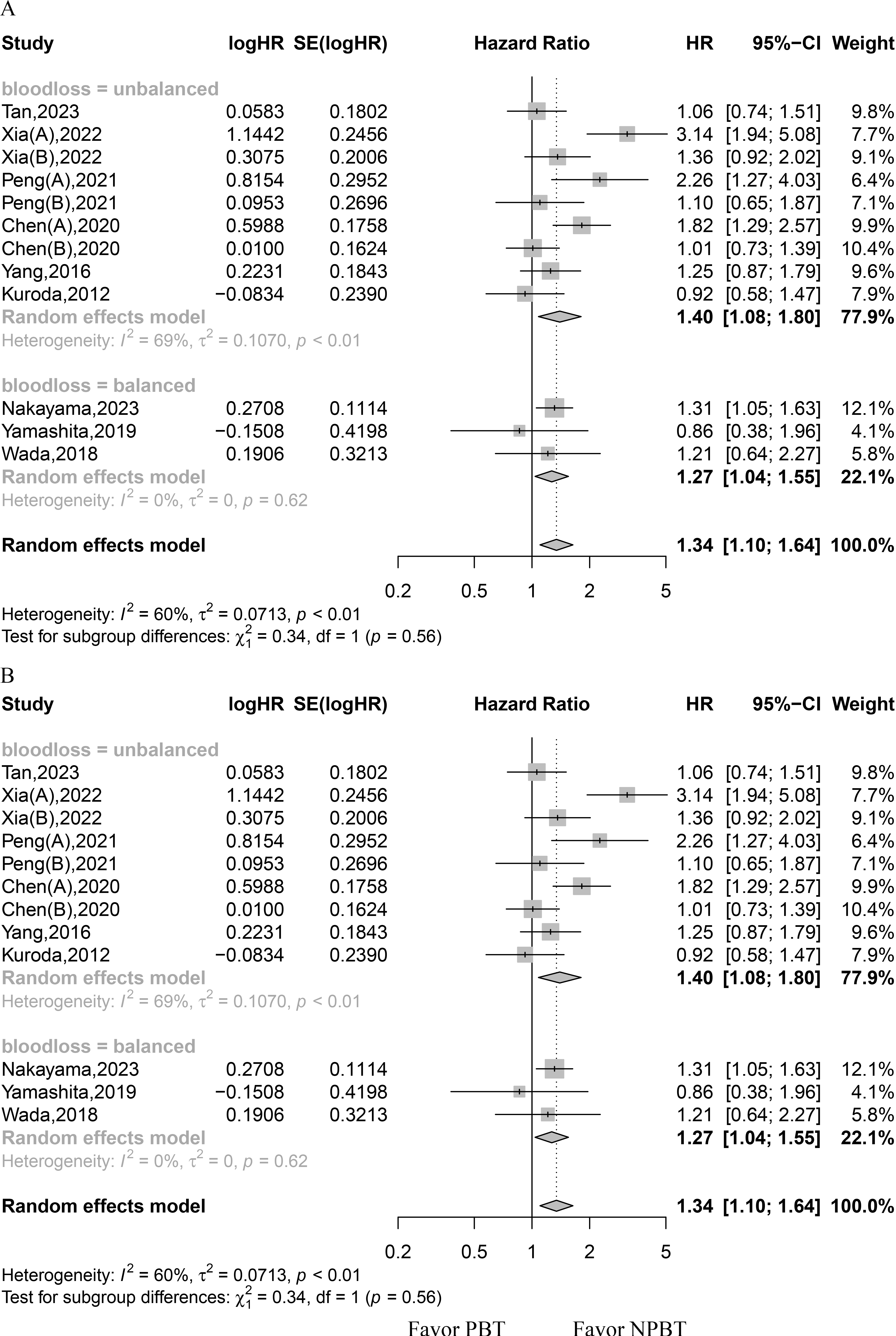


Supplementary material S7 Subgroup analysis based on whether the patients died in early period post operation for overall survival and recurrence-free survival.

A, subgroup analysis based on whether the patients died in early period post operation between two groups in included studies for overall survival; B, subgroup analysis based on whether the patients died in early period post operation for recurrence-free survival.


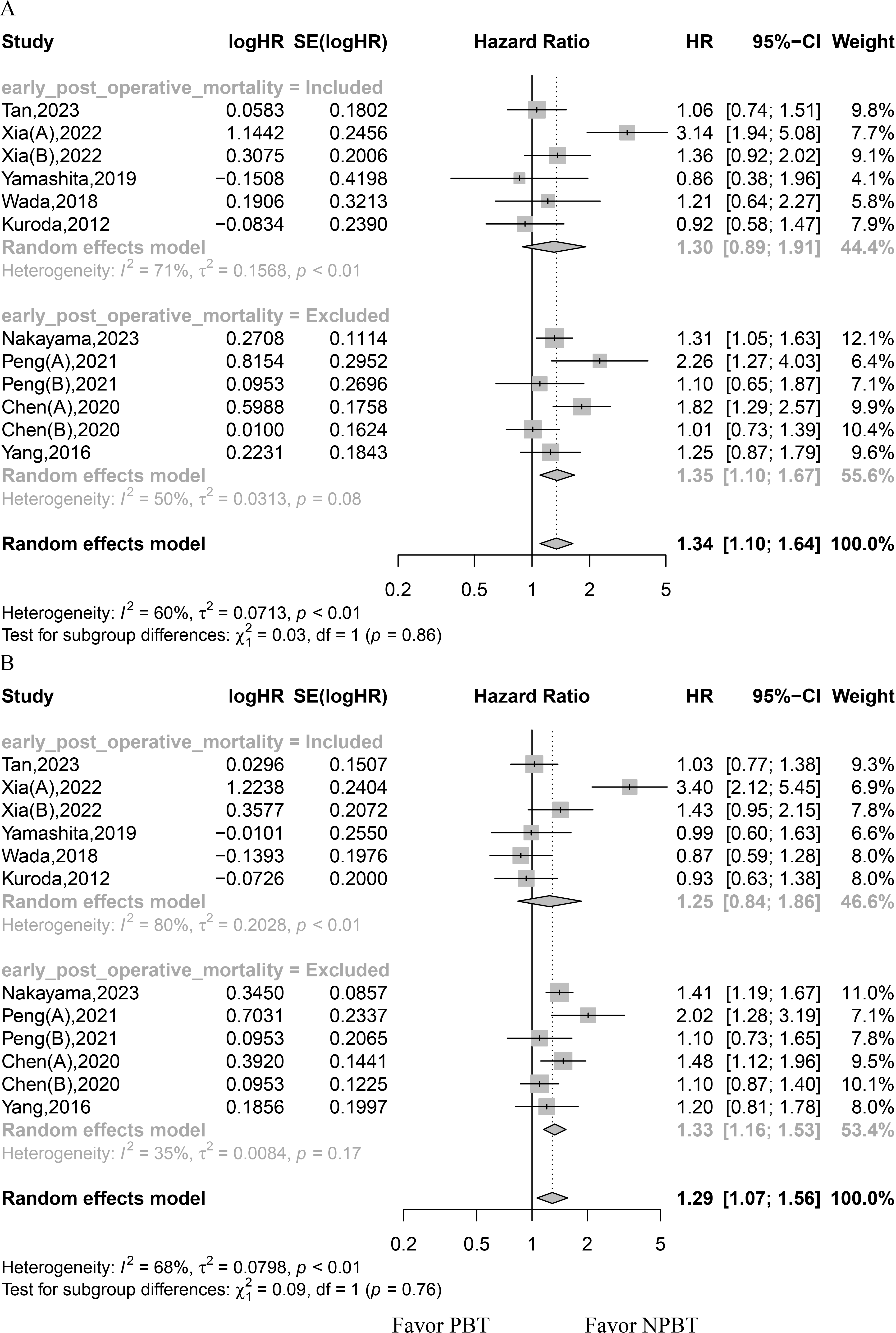

Supplement: Supplementary file 1 [file DataSheet_1.docx]
